# NPM1 Is a Prognostic Biomarker Involved in Immune Infiltration of Lung Adenocarcinoma and Associated With m6A Modification and Glycolysis

**DOI:** 10.3389/fimmu.2021.724741

**Published:** 2021-07-16

**Authors:** Xu-Sheng Liu, Lu-Meng Zhou, Ling-Ling Yuan, Yan Gao, Xue-Yan Kui, Xiao-Yu Liu, Zhi-Jun Pei

**Affiliations:** ^1^Department of Nuclear Medicine and Institute of Anesthesiology and Pain, Taihe Hospital, Hubei University of Medicine, Shiyan, China; ^2^Hubei Key Laboratory of Embryonic Stem Cell Research, Shiyan, China; ^3^Department of Nuclear Medicine, Huanggang Central Hospital, Huanggang, China; ^4^Department of Pathology, Taihe Hospital, Hubei University of Medicine, Shiyan, China

**Keywords:** NPM1, lung adenocarcinoma, immune infiltration, m6A modification, glycolysis

## Abstract

**Background:**

Overexpression of NPM1 can promote the growth and proliferation of various tumor cells. However, there are few studies on the comprehensive analysis of NPM1 in lung adenocarcinoma (LUAD).

**Methods:**

TCGA and GEO data sets were used to analyze the expression of NPM1 in LUAD and clinicopathological analysis. The GO/KEGG enrichment analysis of NPM1 co-expression and gene set enrichment analysis (GSEA) were performed using R software package. The relationship between NPM1 expression and LUAD immune infiltration was analyzed using TIMER, GEPIA database and TCGA data sets, and the relationship between NPM1 expression level and LUAD m6A modification and glycolysis was analyzed using TCGA and GEO data sets.

**Results:**

NPM1 was overexpressed in a variety of tumors including LUAD, and the ROC curve showed that NPM1 had a certain accuracy in predicting the outcome of tumors and normal samples. The expression level of NPM1 in LUAD is significantly related to tumor stage and prognosis. The GO/KEGG enrichment analysis indicated that NPM1 was closely related to translational initiation, ribosome, structural constituent of ribosome, ribosome, Parkinson disease, and RNA transport. GSEA showed that the main enrichment pathway of NPM1-related differential genes was mainly related to mTORC1 mediated signaling, p53 hypoxia pathway, signaling by EGFR in cancer, antigen activates B cell receptor BCR leading to generation of second messengers, aerobic glycolysis and methylation pathways. The analysis of TIMER, GEPIA database and TCGA data sets showed that the expression level of NPM1 was negatively correlated with B cells and NK cells. The TCGA and GEO data sets analysis indicated that the NPM1 expression was significantly correlated with one m6A modifier related gene (HNRNPC) and five glycolysis related genes (ENO1, HK2, LDHA, LDHB and SLC2A1).

**Conclusion:**

NPM1 is a prognostic biomarker involved in immune infiltration of LUAD and associated with m6A modification and glycolysis. NPM1 can be used as an effective target for diagnosis and treatment of LUAD.

## Introduction

Recent studies show that lung adenocarcinoma (LUAD) is the second most diagnosed cancer and the leading cause of cancer death worldwide (1). Despite improved diagnosis and treatment strategies for lung disease, LUAD patients still have a high mortality rate and poor prognosis (2). The development of LUAD is a complex multi-step process, which may be closely related to the abnormal expression of some genes. Therefore, a better understanding of the molecular mechanisms of LUAD could provide more accurate biomarkers for tumor diagnosis and treatment.

Nucleophosmin 1 (NPM1) is a multifunctional protein that is mainly localized in nucleoli and shuttles between the nucleus and cytoplasm (3). In recent years, the focus of NPM1 research has gradually shifted from hematological diseases to solid tumors (4, 5). Previous studies have demonstrated that NPM1 is overexpressed in several types of tumors and promotes the occurrence and progression of tumors (6–8). Our previous studies found high expression of NPM1 in LUAD, but failed to investigate the biological function of NPM1 more broadly (9).

Tumor immunotherapy, N6-methyladenosine (m6A) modification and targeted glycolytic pathway are hot spots in cancer therapy, which have been used for a wide variety of applications in the research and treatment of LUAD. However, there have been few studies on the multifaceted analysis of NPM1 in LUAD, especially the relationship between NPM1 with LUAD immunotherapy, glycolysis and m6A modification.

In this study, we downloaded The Cancer Genome Atlas (TCGA) LUAD data sets and Gene Expression Omnibus (GEO) data sets. Bioinformatics analysis was performed using R software package and other online databases to investigate differences in NPM1 expression in different cancers, and cell assay and immunohistochemistry (IHC) were used to verify differences in NPM1 expression between LUAD samples and normal samples. The NPM1 co-expression gene network in LUAD was analyzed from multiple aspects, and the biological functions and signal transduction pathways of these genes were analyzed. Finally, the relationship between NPM1 and tumor immune cell infiltration, m6A and glycolysis related genes was discussed, which is helpful to understand the possible mechanism of LUAD.

## Materials and Methods

### Ethics Statement

The protocol of this study had been approved by the Ethics Committee of Taihe Hospital Affiliated of Hubei University of Medicine (Shiyan, China) and conducted according to the principles stated in the Declaration of Helsinki.

### Expression of NPM1 in LUAD

We used Oncomine (www.oncomine.org) (10, 11) online database and TCGA data sets (www.tcga-data.nci.nih.gov/tcga) (12) to analyze the difference of NPM1 expression in different tumors. Oncomine database used Student’s t test to compare the expression level of NPM1 in cancer samples and control group, and selected data with fold change > 2 and P value < 0.000001. We also analyzed the LUAD data sets in TCGA (n = 594) and GEO (www.ncbi.nlm.nih.gov/geo; GSE31210, n = 246) (13) data sets to study the difference of NPM1 expression between tumor tissues and normal tissues. The relationship between NPM1 expression level and clinicopathological characteristics of LUAD patients was studied by analyzing the clinical data of LUAD data sets in TCGA database, and the prognostic and diagnostic value of NPM1 in LUAD was evaluated by Cox model and ROC curve. Finally, we verified the differential expression of NPM1 in LUAD and normal samples by qRT-PCR and IHC staining. The specific procedures refer to previous studies (14), and see the **Supplementary Materials** for details.

### Enrichment Analysis of NPM1 Gene Co-Expression Network In LUAD

The TCGA LUAD data sets was analyzed using the stat packet of R software to study the co-expression genes related to NPM1 expression. Pearson’s correlation coefficient was calculated to test the statistical correlation, and ggplot2 package of R software was used to draw volcano map and heat map for display. Gene ontology (GO) function and Kyoto Encyclopedia of Genes and Genomes (KEGG) pathway enrichment analysis of co-expressed genes were performed by clusterProfiler package (version: 3.18.0) (15) of R software, and visual analysis of data was performed by ggplot2 software package.

### Gene Set Enrichment Analysis

To further understand the underlying mechanism of NPM1, we divided samples from the TCGA LUAD data sets into two groups based on the median expression level of NPM1 and performed GSEA (www.gsea-msigdb.org/gsea/index.jsp) (16) to investigate whether genes in the two groups were rich in meaningful biological processes. The annotated gene set c2.cp.v7.2.symbols.gmt [Curated] was selected as the reference gene set. FDR (qvalue) < 0.25 and P < 0.05 were considered statistically significant.

### Correlation Between NPM1 and Tumor Immune Infiltrating Cells

To further explore the potential immunomodulatory mechanism of NPM1 in the regulation of tumor-infiltrating immune cells, we used the TIMER database (www.cistrome.shinyapps.io/timer) (17, 18) to evaluate the correlation between NPM1 expression in TCGA LUAD samples and immune infiltrating cells. Immune infiltrating cells include B cells, neutrophils, CD4+ T cells, macrophages, CD8+ T cells and dendritic cells. We analyzed the relationship between NPM1 copy number variation (CNV) and immune cell infiltration using the somatic copy number alteration (SCNA) module in the TIMER database. R’s CIBERSORT (19) software package was used to detect the proportion of 22 immune cells in LUAD samples with high and low NPM1 expression. We further performed Kaplan-Meier curve analysis to investigate the differences in survival between high and low expression levels of NPM1 and immune cell. In addition, we analyzed the association between NPM1 and immune cell marker genes in LUAD samples using TIMER, GEPIA, and TCGA databases. Immune cell markers are selected from the website of R&D Systems (www.rndsystems.com/cn/resources/cell-markers/immune-cells).

### Correlations of NPM1 Expression With m6A Modification in LUAD

The R software package was used to analyze the correlation between the NPM1 expression and the m6A related genes expression in the GSE31210 and TCGA LUAD data sets, including ZC3H13, YTHDF3, HNRNPA2B1, IGF2BP1, IGF2BP3, YTHDC2, YTHDF1, FTO, HNRNPC, METTL14, METTL3, WTAP, RBM15, ALKBH5, IGF2BP2, RBMX, RBM15B, YTHDC1, VIRMA and YTHDF2 (20). R software package was used to analyze the proportion of m6A related genes in LUAD samples with high and low NPM1 expression. The Kaplan-Meier curve showed the relationship between he expression of related genes and the prognosis of LUAD. The data were analyzed visually by ggplot2 software package.

### Correlations of NPM1 Expression With Glycolysis in LUAD

To further analyze the correlation between NPM1 expression and LUAD glycolysis, R software package was used to analyze the correlation between expression of NPM1 and glycolysis related genes in GSE31210 and TCGA LUAD data sets, including ENO1, G6PD, HK1, HK2, LDHA, LDHB, PDHB, PDK3, PDK4, PGK1, PKM, SLC2A1, SLC2A2 and SLC2A3. The proportion of glycolysis related genes in LUAD samples with high and low NPM1 expression was analyzed by R software package. Kaplan-Meier curves showed the relationship between the expression of related genes and the prognosis of LUAD. The software package ggplot2 was used for visual analysis of the data. To further confirm the idea that NPM1 overexpression affects the glycolysis of LUAD, we retrospectively analyzed images of 40 LUAD patients who underwent ^18^F-FDG PET/CT scans and analyzed them with IHC scores of the corresponding surgically resected tissues to explore the possibility that NPM1 may influence the glycolysis process of LUAD.

## Results

### Pan-Cancer Analysis of NPM1 mRNA Expression in Different Databases

**Figure 1:**
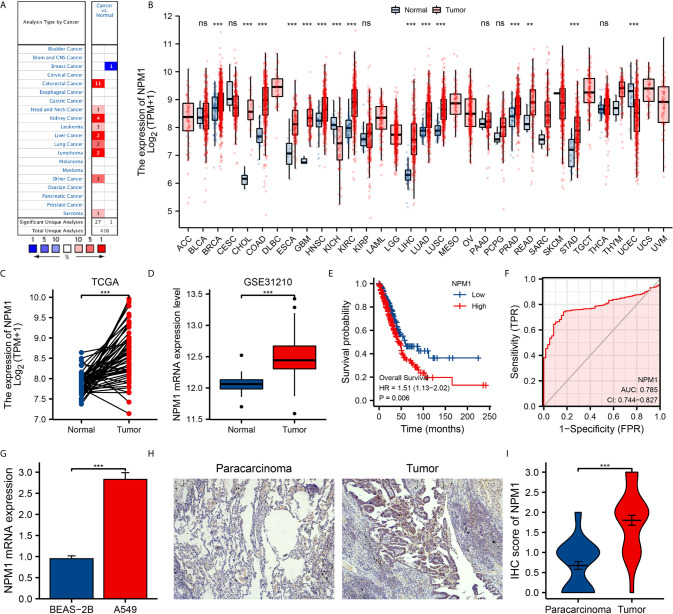
The expression of NPM1 in lung adenocarcinoma (LUAD) and pan-carcinoma. **(A)** NPM1 mRNA expression levels in pan-cancer were measured using Oncomine. **(B)** Pan-cancer data downloaded from the TCGA data sets were used to assess NPM1 mRNA expression levels. **(C)** Difference in expression of NPM1 between LUAD and matched normal tissues in TCGA data sets. **(D)** Difference in expression of NPM1 between LUAD and normal tissues in GSE31210 data sets. **(E)** The survival curve of NPM1. **(F)** ROC curve analysis of NPM1 diagnosis. **(G)** Difference of expression of NPM1 in LUAD cell lines and human normal lung epithelial cell lines. **(H)** Immunohistochemistry assay was used to analyze the expression of NPM1 in LUAD tissues and paracarcinoma tissues. **(I)** The mean NPM1 IHC score in LUAD tissue was significantly higher than that of matched paracarcinoma tissue. *P < 0.05; **P < 0.01; ***P < 0.001; ****P < 0.0001. ns, not significant.

We used Oncomine online database and TCGA data sets to analyze the difference of NPM1 mRNA expression between LUAD group and control group. Oncomine database analysis showed that the expression of NPM1 in colorectal cancer (21–24), head-neck cancer (25), kidney cancer (26–28), leukemia (29), liver cancer (30), lung cancer (31, 32), lymphoma (33) and sarcoma (34) was higher than that in normal tissues. The expression of NPM1 in breast cancer (35) was lower than that in normal tissues (**Figure 1A**). **Table 1** summarizes the details of NPM1 expression in various cancers.

**Table 1:** NPM1 expression in cancerous versus normal tissue in ONCOMINE.

Cancer Site	Cancer Type	P Value	t−Test	Fold Change	Reference (PMID)
Breast	Invasive Breast Carcinoma	1.51E-31	-24.245	-34.469	18438415
Colorectal	Colon Adenocarcinoma	6.36E-9	7.443	2.299	11306497
	Colon Adenoma	2.66E-18	13.932	2.737	18171984
	Cecum Adenocarcinoma	6.08E-15	11.581	3.115	TCGA Colorectal
	Colon Mucinous Adenocarcinoma	6.11E-11	9.054	3.475	TCGA Colorectal
	Rectal Adenocarcinoma	3.11E-17	11.654	2.592	TCGA Colorectal
	Colon Adenocarcinoma	1.10E-17	13.451	2.657	TCGA Colorectal
	Colorectal Carcinoma	5.56E-12	8.599	2.158	20957034
	Colon Adenocarcinoma	5.09E-14	8.859	2.209	17640062
	Colon Adenoma	4.12E-8	10.752	2.714	20957034
	Colon Carcinoma	8.28E-7	9.333	2.185	20957034
	Colorectal Carcinoma	8.03E-14	14.326	3.487	20957034
Head-Neck	Oral Cavity Squamous Cell Carcinoma	4.20E-8	6.277	2.232	21853135
Kidney	Hereditary Clear Cell Renal Cell Carcinoma	1.93E-13	11.212	2.078	19470766
	Non-Hereditary Clear Cell Renal Cell Carcinoma	1.27E-9	7.895	2.034	19470766
	Clear Cell Renal Cell Carcinoma	3.22E-7	8.348	2.604	17699851
	Clear Cell Renal Cell Carcinoma	1.38E-11	9.277	2.245	16115910
Leukemia	Pro-B Acute Lymphoblastic Leukemia	1.65E-11	9.502	2.498	20406941
	T-Cell Acute Lymphoblastic Leukemia	1.24E-25	12.948	2.316	20406941
	Acute Myeloid Leukemia	6.46E-25	14.102	2.043	20406941
Liver	Hepatocellular Carcinoma	2.84E-71	23.638	2.632	21159642
	Hepatocellular Carcinoma	2.28E-8	7.333	2.421	21159642
Lung	Lung Adenocarcinoma	1.28E-7	6.128	2.025	17540040
	Squamous Cell Lung Carcinoma	2.29E-11	9.623	2.262	20421987
Lymphoma	Burkitt’s Lymphoma	8.78E-8	8.028	3.979	18794340
	Diffuse Large B-Cell Lymphoma	2.77E-7	6.644	3.860	18794340
Sarcoma	Myxoid/Round Cell Liposarcoma	6.64E-7	9.520	2.786	20601955

We further analyzed the expression of NPM1 mRNA in human tumors using TCGA data sets. **Figure 1B** shows the difference of NPM1 in different tumor tissues and normal tissues. Compared with normal tissues, the expression level of NPM1 was significantly increased in BRCA (breast invasive carcinoma), CHOL (cholangiocarcinoma), COAD (colon adenocarcinoma), ESCA (esophageal carcinoma), GBM (glioblastoma multiforme), HNSC (head and neck squamous cell carcinoma), KIRC (kidney renal clear cell carcinoma), LIHC (liver hepatocellular carcinoma), LUAD (lung adenocarcinoma), LUSC (lung squamous cell carcinoma), PRAD (prostate adenocarcinoma), READ (rectum adenocarcinoma) and STAD (stomach adenocarcinoma), while it was significantly decreased in KICH (kidney chromophobe) and UCEC (uterine corpus endometrial carcinoma).

### Expression Levels of NPM1 in LUAD Patients

We analyzed LUAD data sets from TCGA and GEO to investigate the differential expression of NPM1 in LUAD samples and normal samples. Analysis of both TCGA and GEO data showed that the expression level of NPM1 was significantly increased in LUAD samples compared to the control group (**Figures 1C, D**). To further prove the accuracy of the predicted results, qRT-PCR and IHC staining experiments were used to further verify the results. qRT-PCR results showed that the expression level of NPM1 mRNA was significantly increased in human lung adenocarcinoma cell lines compared with normal human lung epithelial cells (**Figure 1G**). IHC staining showed that NPM1 was mainly expressed in the nucleus of LUAD cells. The NPM1 IHC score in tumor sample tissue was significantly higher than that in paracancerous tissue (**Figures 1H, I**). These results suggest that NPM1 overexpression may contribute to the progression of LUAD. To further evaluate the prognostic and diagnostic potential of NPM1 in LUAD, we performed Cox regression model and ROC curve analysis. The results of Cox regression model analysis showed that high expression of NPM1 in LUAD predicted worse survival (HR = 1.51(1.13-2.02), P = 0.006) (**Figure 1E**). The results of ROC analysis showed that NPM1 had a good prediction accuracy for LUAD, and the area under the ROC curve was 0.785 (95%CI: 0.744-0.827) (**Figure 1F**).

**Figure 2:**
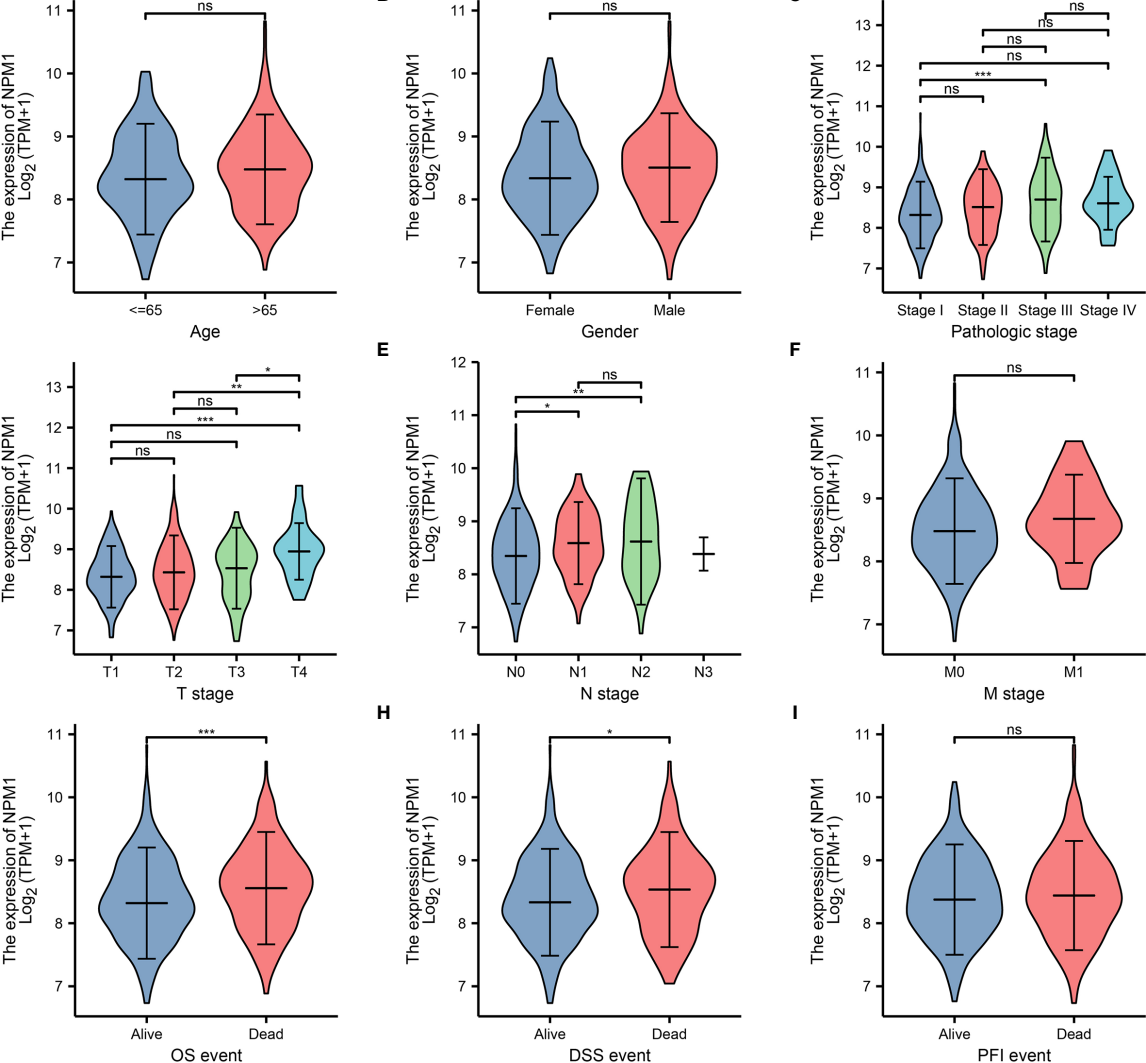
Relationship between NPM1 mRNA expression and clinicopathological parameters in lung adenocarcinoma (LUAD) patients. The NPM1 mRNA expression level was expressed by using ggplot2 package of R software for the patient characteristics of **(A)** age, **(B)** gender, **(C)** pathologic stage, **(D)** T stage, **(E)** N stage, **(F)** M stage, **(G)** OS event, **(H)** DSS event and **(I)** PFI event. *P < 0.05; **P < 0.01; ***P < 0.001; ****P < 0.0001. ns, not significant.

To further determine the potential importance of NPM1 in clinical Settings, we analyzed clinical outcomes from TCGA LUAD samples. The results showed (**Figure 2**) that the expression of NPM1 in Stage II group was significantly higher than that in Stage I group. The expression of NPM1 in T4 group was higher than that in T1, T2 and T3 groups. The expression of NPM1 in N0 group was lower than that in N1 and N2 groups. During OS events, NPM1 expression was significantly higher in patients who died than in the surviving group. Similarly, NPM1 expression was significantly higher in patients who died than in the survival group during DSS events.

### Enrichment Analysis of NPM1 Gene Co-Expression Network in LUAD

**Figure 3:**
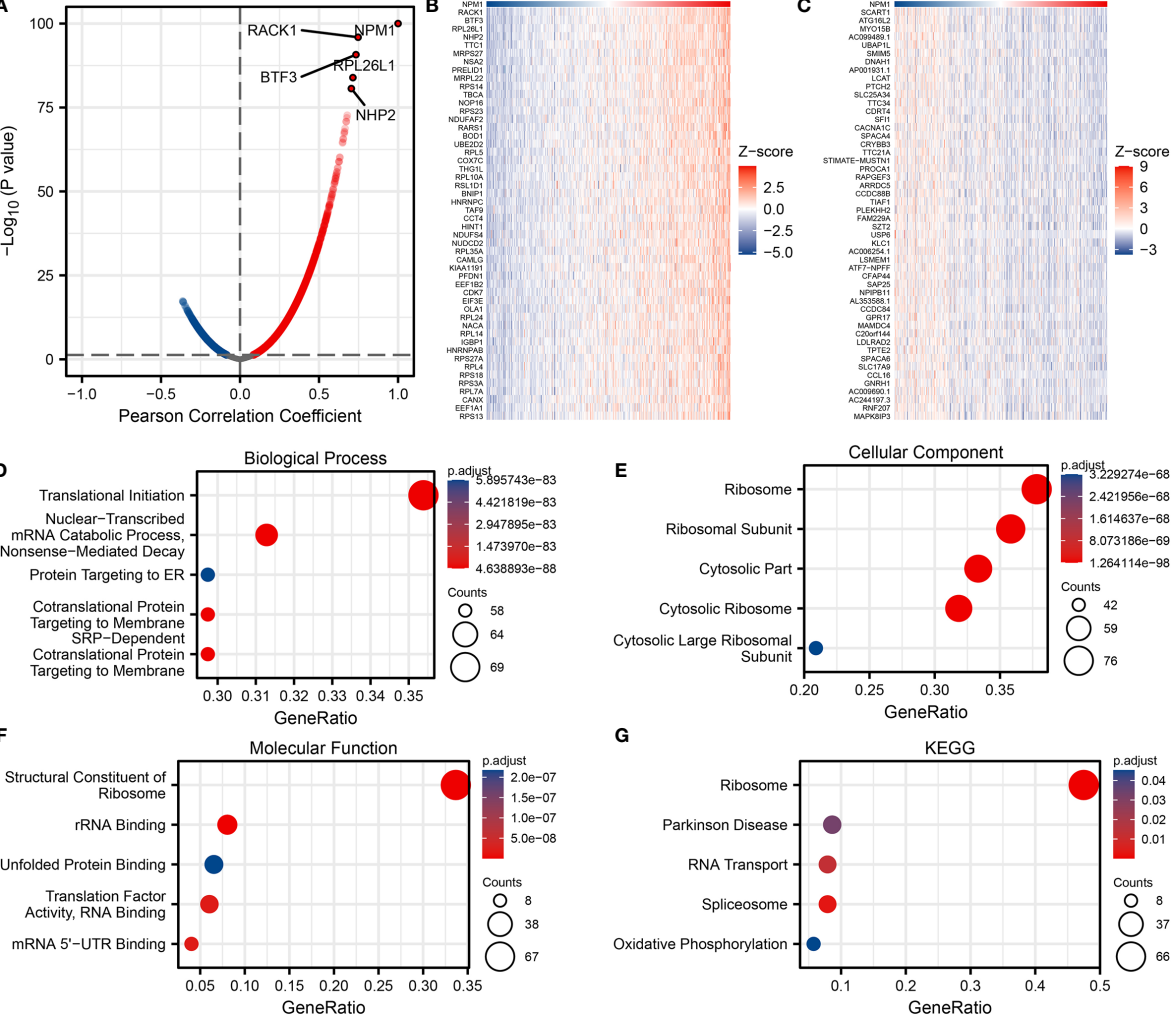
Enrichment analysis of NPM1 gene co-expression network in lung adenocarcinoma (LUAD). **(A)** Volcano map showed co-expression genes associated with NPM1 expression in TCGA LUAD data sets. **(B, C)** Heat maps showed the top 50 co-expression genes positively and negatively correlated with NPM1 expression in the LUAD data sets. **(D–F)** Enrichment analysis of gene ontology (GO) terms for NPM1 co-expression genes. **(G)** Enrichment analysis of Kyoto Encyclopedia of Genes and Genomes (KEGG) terms for terms for NPM1 co-expression genes.

We used the stat package of R software to analyze the co-expressed genes associated with NPM1 expression in the LUAD data sets of TCGA. Only the data of protein-coding genes were retained. As shown in **Figure 3A**, 5845 genes were positively correlated with the expression of NPM1, and 4625 genes were significantly negatively correlated with the expression of NPM1 (P < 0.05). When the threshold selection was cor > 0.7 and P < 0.05, four genes showed the strongest correlation, namely RACK1 (cor = 0.747, P = 1.196E-96), BTF3 (cor = 0.734, P = 1.867E-91), RPL26L1 (cor = 0.714, P = 1.273E-84) and NHP2 (cor = 0.704, P = 2.323E-81). The heat map showed the top 50 important genes positively and negatively correlated with NPM1 expression, respectively (**Figures 3B, C**). The detailed description of co-expressed genes is shown in **Supplementary Table 1**.

The GO function and KEGG pathway enrichment analysis of the top 200 co-expressed genes positively correlated with NPM1 expression were performed by R software package. Under the condition of p.adj < 0.05 and qvalue < 0.2, NPM1 co-expressed genes were involved in 156 biological process (GO-BP), 60 cell component (GO-CC), 16 molecular function (GO-MF) and 5 KEGG. The bubble graph demonstrates the top 5 messages for GO-BP, GO-CC, GO-MF and KEGG, respectively. GO functional annotations showed that NPM1 co-expressed genes were mainly involved in the translational initiation, ribosome, and structural constituent of ribosome (**Figures 3D–F**). KEGG pathway analysis demonstrated that the co-expression of NPM1 was primarily associated to the ribosome, Parkinson disease, and RNA transport (**Figure 3G**). **Supplementary Table 2** summarized the details of the GO function and KEGG pathway of NPM1 co-expression enrichment analysis.

### Gene Set Enrichment Analysis

**Figure 4:**
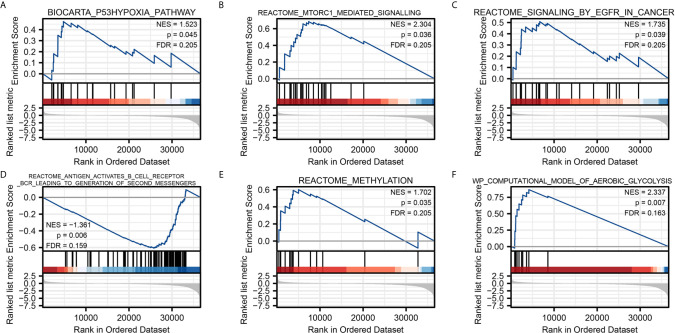
Gene Set Enrichment Analysis. Pathway enriched in the p53 hypoxia pathway **(A)** mTORC1 mediated signaling **(B)** signaling by EGFR in cancer **(C)** antigen activates B cell receptor BCR leading to generation of second messengers **(D)** methylation **(E)** and aerobic glycolysis **(F)**.

To characterize the potential function of NPM1 gene, GSEA was performed on the differential genes. A total of 419 gene sets were found, including mTORC1 mediated signaling (FDR = 0.205, P = 0.036), p53 hypoxia pathway (FDR = 0.205, P = 0.045), signaling by EGFR in cancer (FDR = 0.205, P = 0.039), antigen activates B cell receptor BCR leading to generation of second messengers (FDR = 0.159, P = 0.006), aerobic glycolysis (FDR = 0.163, P = 0.007), methylation (FDR = 0.205, P = 0.035) (**Figure 4**). Detailed enrichment analysis information is shown in **Supplementary Table 3**.

### Correlation Between NPM1 and Tumor Immune Infiltrating Cells

**Figure 5:**
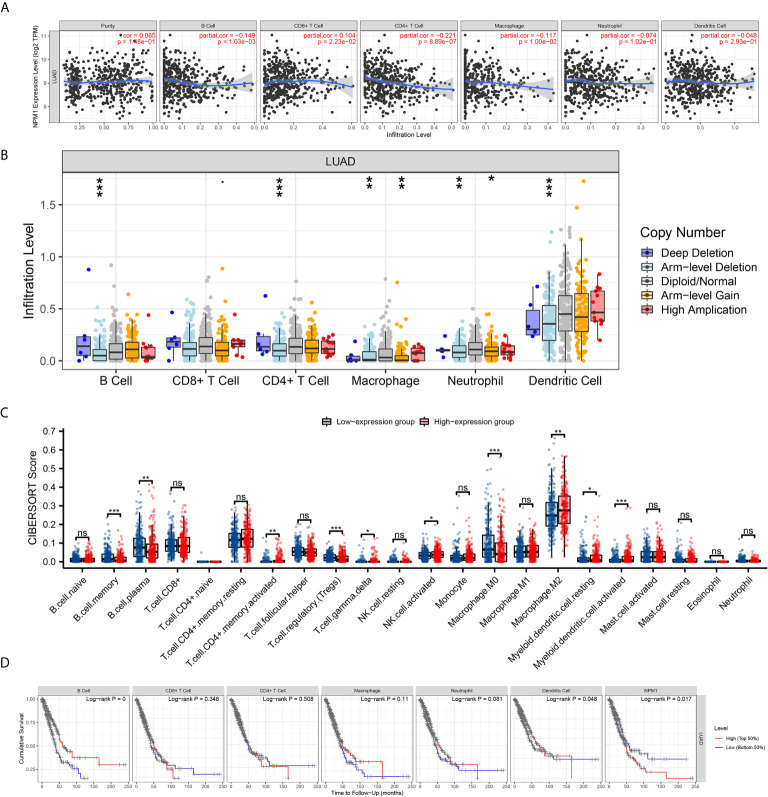
Correlation between NPM1 and Tumor Immune Infiltrating Cells. **(A)** Correlation between the expression of NPM1 and immune infiltrating cells in lung adenocarcinoma (LUAD). **(B)** NPM1 CNV affects the infiltrating levels of B cell, CD4+ T cell, macrophages, neutrophils and dendritic cell in LUAD. **(C)** Changes of 22 immune cell subtypes between high and low NPM1 expression groups in LUAD tumor samples. **(D)** Kaplan-Meier plots of immune infiltration and NPM1 expression levels in LUAD. *P < 0.05; **P < 0.01; ***P < 0.001; ****P < 0.0001. ns, not significant.

We used the TIMER database to analyze the correlation between NPM1 expression and immune infiltrating cells in LUAD. The results showed that the expression of NPM1 was negatively correlated with the expression levels of B cells (r = -0.149, P = 1.03E-3), CD4+ T cell (r = -0.221, P = 8.89E-7) and macrophages (r = -0.117, P = 1.00E-2), while positively correlated with the expression levels of CD8+ T cells (r = 0.104, P = 2.23E-2) (**Figure 5A**). At the same time, we found that NPM1 CNV has a closely association with the degree of infiltration of B cell, CD4+ T cell, macrophages, neutrophils and dendritic cell (**Figure 5B**).

CIBERSORT analysis showed that NPM1 expression level had correlation with tumor immune cell infiltration (**Figure 5C**), including B cell memory (P < 0.001), B cell plasma (P = 0.003), T cell CD4+ memory activated (P = 0.004), T cell regulatory (Tregs) (P < 0.001), T cell gamma delta (P = 0.031), NK cell activated (P = 0.036), Macrophage M0 (P < 0.001), Macrophage M2 (P = 0.007), Myeloid dendritic cell resting (P = 0.032) and Myeloid dendritic cell activated (P < 0.001). We further generated Kaplan-Meier curve using the TIMER database to investigate the differences in survival between high and low expression levels of NPM1 and immune cell. We found B cell infiltration (P < 0.001), dendritic cell infiltration (P = 0.048) and NPM1 expression (P= 0.017) to significantly correlate with LUAD prognosis (**Figure 5D**).

**Figure 6:**
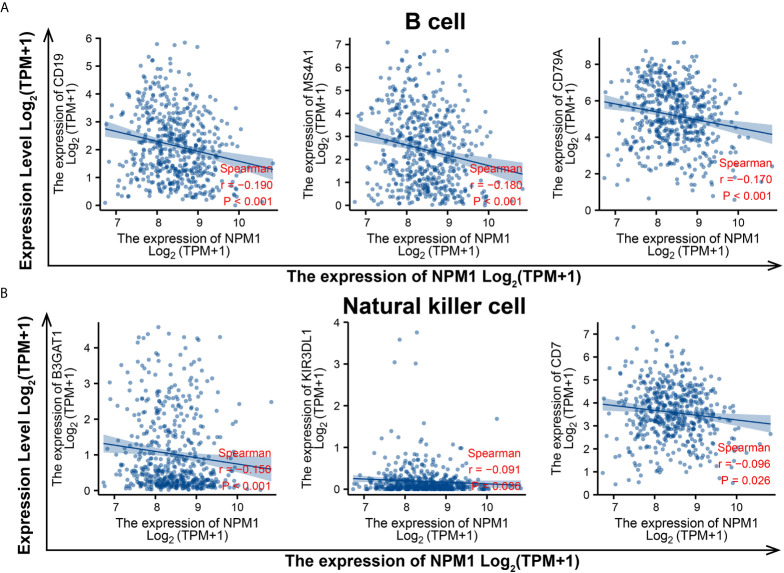
NPM1 expression correlated with B cell and natural killer cell in lung adenocarcinoma (LUAD). Markers include CD19, MS4A1 and CD79A of B cell **(A)** B3GAT1, KIR3DL1 and CD7 of natural killer cell **(B)**.

To evaluate the relationship between NPM1 and various immune infiltrating cells of LUAD, TIMER, GEPIA databases and TCGA LUAD data sets were analyzed to analyze the association between NPM1 and immune marker genes of several immune cells (**Table 2**). All three analyses demonstrated that the expression of NPM1 was associated with B cell and NK cell immune marker genes, including CD19, MS4A1, CD79A, B3GAT1, KIR3DL1 and CD7. The scatter plot showed the correlation between NPM1 expression and B cell and NK cell immune marker genes, respectively (**Figure 6**).

**Table 2:** Correlation analysis between NPM1 and immune cell marker gene in TIMER, GEPIA and TCGA.

Description	Gene markers	TIMER		GEPIA		TCGA	
		Purity		Tumor		Tumor	
		rho	P	rho	P	rho	P
B cell	CD19	-0.197	**1.08E-05**	-0.24	**5.00E-08**	-0.193	**6.86E-06**
	MS4A1	-0.149	**9.01E-04**	-0.18	**6.70E-05**	-0.184	**1.88E-05**
	CD79A	-0.189	**2.39E-05**	-0.26	**1.20E-08**	-0.169	**8.43E-05**
CD8+ T Cell	CD8A	0.013	7.69E-01	0.0061	8.90E-01	0.048	2.67E-01
	CD8B	-0.013	7.68E-01	-0.016	7.30E-01	0.037	3.99E-01
	IL2RA	0.099	**2.81E-02**	0.15	**1.20E-03**	0.111	**1.04E-02**
Tfh	CXCR3	-0.134	**2.79E-03**	-0.14	**2.70E-03**	-0.050	2.51E-01
	CXCR5	-0.168	**1.83E-04**	-0.39	**3.50E-09**	-0.130	**2.63E-03**
	ICOS	-0.006	8.89E-01	0.019	6.70E-01	-0.023	5.99E-01
Th1	IL12RB1	-0.103	**2.23E-02**	-0.082	7.30E-02	-0.070	1.04E-01
	CCR1	-0.019	6.69E-01	0.05	2.70E-01	0.045	2.97E-01
	CCR5	-0.037	4.11E-01	0.0054	9.10E-01	-0.019	6.56E-01
Th2	CCR4	-0.031	4.93E-01	0.02	6.60E-01	-0.061	1.56E-01
	CCR8	0.029	5.18E-01	0.091	**4.60E-02**	0.023	6.03E-01
	HAVCR1	0.080	7.72E-02	0.088	5.20E-02	0.046	2.93E-01
Th17	IL21R	-0.087	5.23E-02	-0.064	1.60E-01	-0.079	6.81E-02
	IL23R	0.012	7.97E-01	0.097	**3.30E-02**	-0.088	**4.15E-02**
	CCR6	-0.095	**3.43E-02**	-0.0089	8.50E-01	-0.103	**1.73E-02**
Treg	FOXP3	-0.054	2.35E-01	-0.057	2.10E-01	0.011	8.00E-01
	NT5E	0.104	**2.12E-02**	0.16	**3.20E-04**	0.162	**1.72E-04**
	IL7R	-0.008	8.53E-01	0.022	6.30E-01	-0.067	1.21E-01
T cell exhaustion	PDCD1	-0.071	1.15E-01	-0.087	5.50E-02	-0.018	6.74E-01
	CTLA4	-0.067	1.39E-01	-0.089	5.20E-02	-0.091	**3.49E-02**
	LAG3	-0.141	**1.74E-03**	-0.19	**2.50E-05**	-0.072	9.61E-02
M1 Macrophage	NOS2	-0.080	7.50E-02	-0.008	8.60E-01	0.007	8.81E-01
	IRF5	-0.223	**5.80E-07**	-0.14	**1.40E-03**	-0.095	**2.83E-02**
	PTGS2	-0.051	2.56E-01	-0.053	2.50E-01	-0.060	1.68E-01
M2 Macrophage	CD163	0.009	8.38E-01	0.048	2.90E-01	0.036	4.09E-01
	MRC1	0.011	8.14E-01	0.12	**1.00E-02**	0.029	5.03E-01
	CD209	0.016	7.15E-01	0.11	**2.10E-02**	0.052	2.30E-01
TAM	CCL2	-0.001	9.74E-01	0.0077	8.70E-01	0.071	1.02E-01
	CD86	-0.021	6.36E-01	0.053	2.40E-01	0.046	2.87E-01
	CD68	-0.055	2.24E-01	0.088	5.30E-02	0.034	4.30E-01
Monocyte	CD14	-0.095	**3.41E-02**	-0.046	3.20E-01	0.050	2.50E-01
	CD33	-0.062	1.66E-01	-0.0073	8.70E-01	0.005	9.12E-01
	ITGAX	-0.197	**9.99E-06**	-0.17	**1.60E-04**	-0.179	**3.32E-05**
Natural killer cell	B3GAT1	-0.138	**2.11E-03**	-0.12	**6.70E-03**	-0.153	**3.79E-04**
	KIR3DL1	-0.158	**4.36E-04**	-0.11	**2.10E-02**	-0.091	**3.62E-02**
	CD7	-0.205	**4.28E-06**	-0.23	**3.50E-07**	-0.096	**2.57E-02**
Neutrophil	FCGR3A	0.034	4.56E-01	0.096	**3.50E-02**	0.092	**3.36E-02**
	CD55	-0.059	1.94E-01	0.058	2.00E-01	0.053	2.21E-01
	ITGAM	-0.090	**4.46E-02**	-0.029	5.20E-01	-0.038	3.81E-01
Dendritic cell	CD1C	-0.069	1.27E-01	-0.008	8.60E-01	0.012	7.77E-01
	THBD	-0.010	8.22E-01	0.087	5.60E-02	0.065	1.33E-01
	NRP1	0.019	6.80E-01	0.12	**9.40E-03**	0.040	3.52E-01

Bold values indicate P < 0.05.

### Correlations of NPM1 Expression With m6A Modification in LUAD

**Figure 7:**
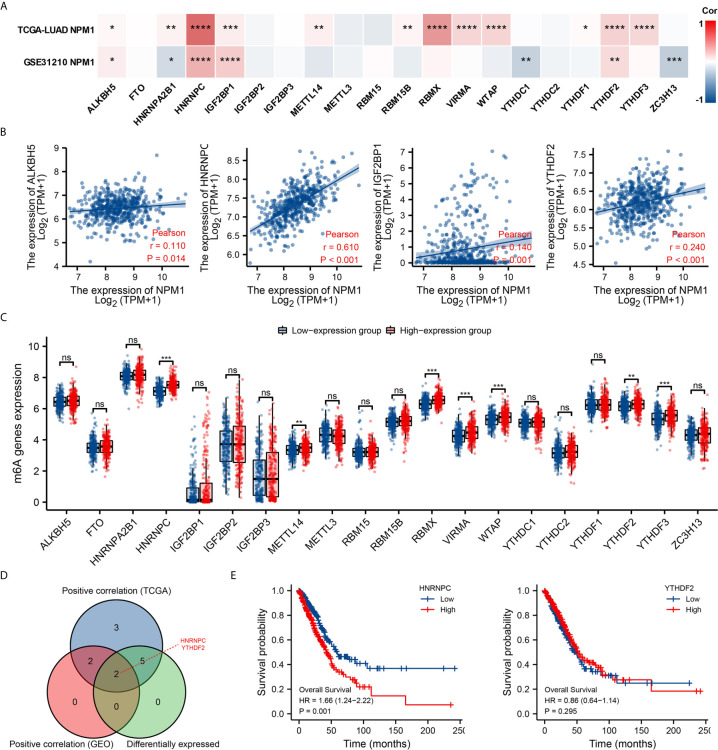
Correlations of NPM1 expression with m6A related genes in lung adenocarcinoma (LUAD). **(A)** GSE31210 and TCGA LUAD data sets analyzed the correlation between the NPM1 and the m6A related genes expression in LUAD. **(B)** Draw a scatter plot to show the correlation between the NPM1 and the m6A related genes expression, include ALKBH5, HNRNPC, IGF2BP1 and YTHDF2. **(C)** The differential expression of m6A related genes between high and low NPM1 expression groups in LUAD tumor samples. **(D)** Venn diagram showed both expression correlation and differential expression of genes, including HNRNPC and YTHDF2. **(E)** Kaplan-Meier curve of HNRNPC and YTHDF2. *P < 0.05; **P < 0.01; ***P < 0.001; ****P < 0.0001. ns, not significant.

Modification of m6A plays an important role in the development of LUAD. By analyzing the GSE31210 and TCGA LUAD data sets to investigate the correlation between NPM1 expression and the expression of 20 m6A related genes in LUAD. The results demonstrated that in the GSE31210 and TCGA LUAD data sets, the expression of NPM1 was significantly positively correlated with ALKBH5, HNRNPC, IGF2BP1 and YTHDF2 (**Figure 7A**, P < 0.05). In addition, NPM1 expression was significantly positively correlated with HNRNPA2B1, METTL14, RBM15B, RBMX, VIRMA, WTAP, YTHDF1 and YTHDF3 in the TCGA LUAD data sets (P < 0.05), while NPM1 expression was negatively correlated with HNRNPA2B1, YTHDC1 and ZC3H13 expression in the GSE31210 data sets (P < 0.05).

The scatter plot shows the association between NPM1 and m6A related genes expression (**Figure 7B**). At the same time, TCGA LUAD samples were divided into high and low expression groups according to the expression level of NPM1. We attempted to analyze the m6A related genes differential expression between high and low groups with NPM1 expression to determine whether m6A modification was different between high and low groups with NPM1 expression in LUAD (**Figure 7C**). The results demonstrated that compared with the low expression group, the expressions of HNRNPC, METTL14, RBMX, VIRMA, WTAP, YTHDF2 and YTHDF3 in the high expression group of NPM1 were increased (P < 0.05). Venn diagram showed both expression correlation and differential expression of genes, including HNRNPC and YTHDF2 (**Figure 7D**). Kaplan-Meier curve showed that high expression of HNRNPC was strongly associated with poor prognosis of LUAD (P = 0.001), while YTHDF2 expression was not associated with poor prognosis of LUAD (P = 0.295) (**Figure 7E**). These results suggest that NPM1 may be closely related to the m6A modification of LUAD, especially through its regulation with HNRNPC, and ultimately affect the progression and prognosis of LUAD.

### Correlations of NPM1 Expression With Glycolysis in LUAD

**Figure 8:**
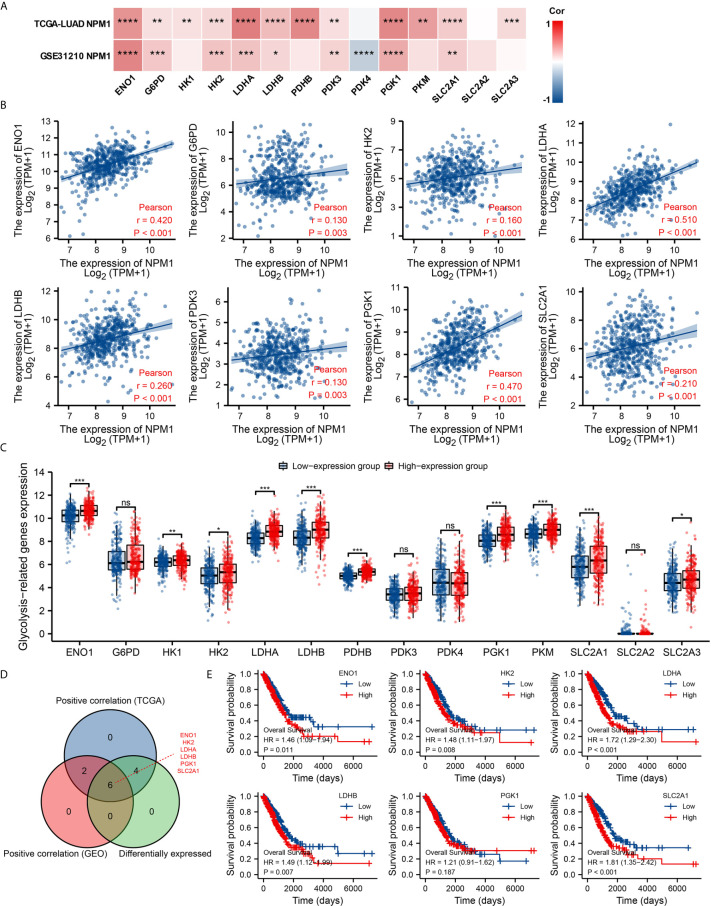
Correlations of NPM1 expression with glycolysis related genes in lung adenocarcinoma (LUAD). **(A)** GSE31210 and TCGA LUAD data sets analyzed the correlation between the NPM1 and the m6A related genes expression in LUAD. **(B)** Draw a scatter plot to show the correlation between the NPM1 and the glycolysis related genes expression, include ENO1, G6PD, HK2, LDHA, LDHB, PDK3, PGK1 and SLC2A1. **(C)** The differential expression of glycolysis related genes between high and low NPM1 expression groups in LUAD tumor samples. **(D)** Venn diagram showed both expression correlation and differential expression of genes, including ENO1, HK2, LDHA, LDHB, PGK1 and SLC2A1. **(E)** Kaplan-Meier curve of ENO1, HK2, LDHA, LDHB, PGK1 and SLC2A1. *P < 0.05; **P < 0.01; ***P < 0.001; ****P < 0.0001. ns, not significant.

Glycolysis of tumor cells plays an important role in the progression of LUAD. By analyzing the GSE31210 and TCGA LUAD data sets to investigate the correlation between NPM1 and the expression of 14 glycolysis related genes in LUAD. The results showed that the expression of NPM1 was significantly positively correlated with ENO1, G6PD, HK2, LDHA, LDHB, PDK3, PGK1 and SLC2A1 in the GSE31210 and TCGA LUAD data sets (**Figure 8A**, P < 0.05). In addition, NPM1 expression was significantly positively correlated with HK1, PDHB, PKM and SLC2A3 in the TCGA LUAD data sets (P < 0.05), while NPM1 expression was negatively correlated with PDK4 expression in the GSE31210 data sets (P < 0.05).

The scatter plot shows the association between NPM1 and glycolysis related genes (**Figure 8B**). At the same time, we attempted to analyze the differential expression of glycolysis related genes between the high and low groups with NPM1 expression (**Figure 8C**). The results demonstrated that compared with the low expression group, the expression of ENO1, HK1, HK2, LDHA, LDHB, PDHB, PGK1, PKM, SLC2A1 and SLC2A3 were increased in the high expression group of NPM1 (P < 0.05). Venn diagram showed both expression correlation and differential expression of genes, including ENO1, HK2, LDHA, LDHB, PGK1 and SLC2A1 (**Figure 8D**). Kaplan-Meier curves showed that high expression of ENO1, HK2, LDHA, LDHB and SLC2A1 was strongly associated with poor prognosis in LUAD (P < 0.05), while PGK1 expression was not (P > 0.05) (**Figure 8E**).

**Figure 9:**
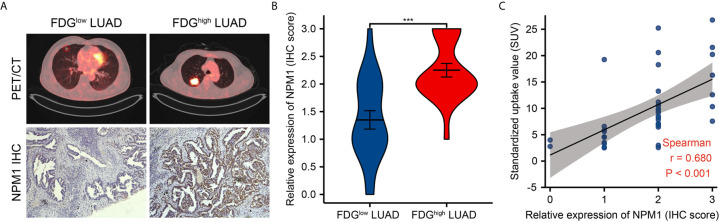
Correlations of NPM1 expression with glycolytic metabolism in lung adenocarcinoma (LUAD). **(A)** Representative PET/CT images and NPM1 immunohistochemical images of LUAD patients with FDG high uptake and FDG low uptake (SUVmax). **(B)** Statistical analysis of NPM1 expression in LUAD patients with FDG high uptake and patients with FDG low uptake. **(C)** Correlation between FDG uptake and NPM1 expression in 40 LUAD patients. *P < 0.05; **P < 0.01; ***P < 0.001; ****P < 0.0001.

Further analysis showed a significant correlation between FDG uptake and NPM1 immunohistochemical staining in LUAD patients (**Figure 9**, P < 0.05). These results suggest that NPM1 may be closely related to the glycolysis of LUAD, especially through the regulation of ENO1, HK2, LDHA, LDHB and SLC2A1, and ultimately affect the progression and prognosis of LUAD.

## Discussion

NPM1 is a highly conserved protein commonly found in eukaryotic cells. It is mainly localized in the nucleus and can shuttle between the nucleus and cytoplasm to participate in nucleocytoplasmic signal transport (3, 4). Studies have shown that the content of NPM1 in tumor cells and growing cells is significantly higher than that in quiescent cells (36, 37). Overexpression of NPM1 can promote the growth and proliferation of various tumor cells (5–8). These results suggest that NPM1 may be a potential target for tumor gene therapy. However, there are few studies on the comprehensive analysis of NPM1 in LUAD.

In the present study, the NPM1 expression in tumors was predicted by bioinformatics analysis, and the expression of NPM1 in LUAD was verified by cell assay and immunohistochemical staining. Through the analysis of Oncomine database, we found NPM1 was overexpressed in 9 types of cancer, and analysis of the TCGA data set found that NPM1 was overexpressed in 13 types of cancer, which was consistent with the results of previous studies (4, 36, 37). Based on the analysis of GEO and TCGA LUAD data sets, the expression level of NPM1 in LUAD tissues was significantly higher than that in normal tissues. The expression of NPM1 in LUAD and normal samples was detected by qRT-PCR and IHC, and the analysis results were consistent with the above results. We also used ROC curve to analyze the ability of NPM1 expression to predict LUAD, and found that NPM1 had certain accuracy in predicting the outcome of tumors and normal samples. Previous studies have found that NPM1 expression had certain accuracy in predicting the prognosis of gastric cancer (38) and prostate cancer (39). At the same time, we also found that high expression of NPM1 predicted a worse prognosis in patients with LUAD, suggesting that changing the expression level of NPM1 may improve the prognosis in patients with LUAD. Finally, NPM1 expression was found to be related to tumor grade. In conclusion, NPM1 may serve as a potential diagnostic and prognostic marker for LUAD.

However, current studies on the role of NPM1 in tumor mainly focus on its role in ribosome processing and assembly, centrosome replication and molecular chaperone (4, 36, 37). Other biological functions of NPM1 in LUAD are less studied. In this study, R software package was used to analyze the co-expression genes of NPM1 in LUAD, and it was found that the expressions of RACK1, BTF3, RPL26L1 and NHP2 in LUAD had the strongest correlation with NPM1. Wu et al. (40) found that PHB2 promotes tumorigenesis *via* RACK1 in non-small cell lung cancer. Jeon et al. (41) found that kahweol inhibited the proliferation of NSCLC cells through ERK-mediated signaling pathways and the downregulation of BTF3, while the role of RPL26L1 and NHP2 in LUAD has not been reported. The GO and KEGG function enrichment analysis of 200 co-expressed genes positively correlated with NPM1 expression demonstrated that the co-expression of NPM1 was primarily associated to translational initiation, ribosome, and structural constituent of ribosome. KEGG pathway analysis showed that the co-expression of NPM1 was primarily associated to ribosome, Parkinson disease, and RNA transport, which was like the findings of previous studies (4). The GSEA pathway enrichment analysis showed that the differential genes grouped according to NPM1 expression were mainly enriched in the mTORC1 mediated signaling, p53 hypoxia pathway, signaling by EGFR in cancer, antigen activates B cell receptor BCR leading to generation of second messengers, aerobic glycolysis and methylation pathways. Previous studies have shown that the occurrence and development of LUAD are closely related to the first three pathways (42–44).

Immune infiltration of tumor cells is associated with lymph node metastasis and prognosis of LUAD (45, 46). TIMER database analysis showed that the expression level of NPM1 in LUDA was negatively correlated with B cells, CD4+ T cells and macrophages, and positively correlated with the expression level of CD8+ T cells. In addition, NPM1 CNV was significantly correlated with the infiltration levels of B cells, CD4+ T cells, macrophages, neutrophils and dendritic cells. These results suggest that NPM1 may be involved in the immune response to the tumor microenvironment of LUAD, especially to B cells, CD4+T cells and macrophages. The proportion of 22 tumor immune cells in LUAD was determined by CIBERSORT analysis. We identified 10 types of immune cells, including memory B cells, plasma B cells, activated memory CD4+ T cells, regulatory T cells, gamma delta T cells, activated NK cells, M0 macrophages, M2 macrophages, resting myeloid dendritic cells and activated myeloid dendritic cell, and their expression ratio showed significant differences with different expression levels of NPM1. At the same time, survival analysis also found that LUAD patients with B cell low expression group had a worse prognosis. In addition, through the analysis of TIMER, GEPIA database and TCGA data sets, we found that the expression of NPM1 was significantly negatively correlated with the gene markers of B cells and NK cells, suggesting that NPM1 may affect the immune infiltration of LUAD by affecting the expression of B cells and NK cells. B cells and NK cells are important immune cells of the body, which have a wide range of anti-tumor effects (47–50). Yang et al. (48) found that in lung cancer cells, blocking the transforming growth factor-β signaling pathway enhanced the antitumor effect of NK-92 cell therapy. Germain et al. (49) found that lung cancer patients with high density B cells had a better prognosis. We speculate that the overexpression of NPM1 inhibits the infiltration of B cells and NK cells in LUAD, and ultimately further accelerates tumor progression. We suggest that the high expression of NPM1 in LUAD patients may trigger an anti-tumor immune response, suggesting that NPM1 plays an important role in the immune regulation of LUAD. However, more experiments are needed to further verify our hypothesis, especially the relationship between NPM1 and B cells and NK cells, respectively.

As a part of methylation modification, m6A modification is one of the most common RNA methylation modifications, which can influence the occurrence and development of cancer by regulating cancer-related biological functions (2, 51, 52). Li et al. (51) found that FTO, as an m6A demethylase, is highly expressed in acute myeloid leukemia and plays an important role in carcinogenesis. However, there are few studies on the relationship between NPM1 and m6A in solid tumors. In this study, we found that the expression level of NPM1 was significantly positively correlated with ALKBH5, HNRNPC, IGF2BP1 and YTHDF2. We also found that the expression levels of HNRNPC, METTL14, RBMX, VIRMA, WTAP, YTHDF2 and YTHDF3 were significantly increased in the high NPM1 expression group. Finally, Kaplan-Meier curve analysis showed that LUAD patients with high HNRNPC expression had a worse prognosis. We believe that the cancer promoting effect of NPM1 gene is related to the modification of m6A, which may affect the methylation level of LUAD through its association with HNRNPC, and ultimately affect the progression of LUAD.

The enhancement of glycolysis is strongly associated to the development of cancer and the poor prognosis. Targeting cancer glycolysis metabolism is a new strategy for cancer treatment (53). Zhu et al. (54) found that NPM1 promoted aerobic glycolysis and tumor progression in patients with pancreatic cancer by inhibiting the fructose-1, 6-bisphosphatase 1. In this study, we found that the expression level of NPM1 was significantly positively correlated with ENO1, G6PD, HK2, LDHA, LDHB, PDK3, PGK1 and SLC2A1.We also found that the expression levels of ENO1, HK1, HK2, LDHA, LDHB, PDHB, PGK1, PKM, SLC2A1 and SLC2A3 were significantly increased in the high expression group of NPM1. Finally, Kaplan-Meier curve analysis showed that LUAD patients with high expression of ENO1, HK2, LDHA, LDHB and SLC2A1 had a worse prognosis. Further analysis found a significant association between FDG uptake and NPM1 immunohistochemical staining in LUAD patients. We suggest that NPM1 may enhance the glycolytic ability of LUAD by promoting the expression of ENO1, HK2, LDHA, LDHB and SLC2A1, and thus promote the occurrence and development of LUAD.

In conclusion, our study confirmed that NPM1 is overexpressed in LUAD, and its expression level is related to clinical case characteristics and prognosis of LUAD patients. The expression level of NPM1 is closely related to the extent of immune cell infiltration, which may reduce the anti-tumor effect by inhibiting the infiltration of B cells and NK cells. NPM1 is associated with m6A modification and glycolysis, and m6A modification may promote the glycolysis and malignant proliferation of LUAD by enhancing the stability of NPM1. NPM1 can be used as a biomarker for the diagnosis, treatment and prognosis of LUAD.

## Data Availability Statement

The datasets presented in this study can be found in online repositories. The names of the repository/repositories and accession number(s) can be found in the article/**Supplementary Material**.

## Ethics Statement

The studies involving human participants were reviewed and approved by The Ethics Committee of Taihe Hospital Affiliated of Hubei University of Medicine. Written informed consent for participation was not required for this study in accordance with the national legislation and the institutional requirements.

## Author Contributions

X-SL conceived the project and wrote the manuscript. X-SL, L-MZ, L-LY and YG participated in data analysis. X-SL, X-YK and X-YL participated in discussion and language editing. Z-JP reviewed the manuscript. All authors contributed to the article and approved the submitted version.

## Funding

This work was supported by the Hubei province’s Outstanding Medical Academic Leader program, the Foundation for Innovative Research Team of Hubei Provincial Department of Education T2020025, the Hubei Provincial Department of Science and Technology Innovation Group Program (grant no. 2019CFA034), Free-exploring Foundation of Hubei University of Medicine (grant no. FDFR201903), Innovative Research Program for Graduates of Hubei University of Medicine (grant no. YC2020011) and the Key Discipline Project of Hubei University of Medicine.

## Conflict of Interest

The authors declare that the research was conducted in the absence of any commercial or financial relationships that could be construed as a potential conflict of interest.

## Supplementary Material

Supplementary Table 1 NPM1 co-expressed genes.

Supplementary Table 2 The GO and KEGG enrichment analysis of NPM1 coexpression genes.
