# Corrigendum: NPM1 Is a Prognostic Biomarker Involved in Immune Infiltration of Lung Adenocarcinoma and Associated With m6A Modification and Glycolysis

**DOI:** 10.3389/fimmu.2021.751004

**Published:** 2021-08-31

**Authors:** Xu-Sheng Liu, Lu-Meng Zhou, Ling-Ling Yuan, Yan Gao, Xue-Yan Kui, Xiao-Yu Liu, Zhi-Jun Pei

**Affiliations:** ^1^Department of Nuclear Medicine and Institute of Anesthesiology and Pain, Taihe Hospital, Hubei University of Medicine, Shiyan, China; ^2^Hubei Key Laboratory of Embryonic Stem Cell Research, Shiyan, China; ^3^Department of Nuclear Medicine, Huanggang Central Hospital, Huanggang, China; ^4^Department of Pathology, Taihe Hospital, Hubei University of Medicine, Shiyan, China

**Keywords:** NPM1, lung adenocarcinoma, immune infiltration, m6A modification, glycolysis

In the original article, there was a mistake in the legend for **Supplementary Table 1**, “HK2 co-expressed genes”, as published. “HK2” was written instead of “NPM1”. The correct legend appears below.

In the original article, there was a mistake in the legend for **Supplementary Table 2**, “The GO and KEGG enrichment analysis of HK2 co-expression genes”, as published. “HK2” was written instead of “NPM1”. The correct legend appears below.

In the original article, there was an error in the section **Abstract, Results**, Paragraph 1. The word “YTHDF2” in this sentence is incorrect: “The TCGA and GEO data sets analysis indicated that the NPM1 expression was significantly correlated with one m6A modifier related gene (YTHDF2) and five glycolysis related genes (ENO1, HK2, LDHA, LDHB and SLC2A1)”.

A correction has been made to the above sentence, as follows: “The TCGA and GEO data sets analysis indicated that the NPM1 expression was significantly correlated with one m6A modifier related gene (HNRNPC) and five glycolysis related genes (ENO1, HK2, LDHA, LDHB and SLC2A1)”.

In the original article, there was an error in the section **Results**, **Correlations of NPM1 Expression With m6A Modification in LUAD**, Paragraph 2. The words, “YTHDF2” and “HNRNPC” in this passage is incorrect: “Kaplan-Meier curve showed that high expression of YTHDF2 was strongly associated with poor prognosis of LUAD (P = 0.001), while HNRNPC expression was not associated with poor prognosis of LUAD (P = 0.295) (**Figure 7E**). These results suggest that NPM1 may be closely related to the m6A modification of LUAD, especially through its regulation with YTHDF2, and ultimately affect the progression and prognosis of LUAD”.

A correction has been made to the above sentence, as follows: “Kaplan-Meier curve showed that high expression of HNRNPC was strongly associated with poor prognosis of LUAD (P = 0.001), while YTHDF2 expression was not associated with poor prognosis of LUAD (P = 0.295) (**Figure 7E**). These results suggest that NPM1 may be closely related to the m6A modification of LUAD, especially through its regulation with HNRNPC, and ultimately affect the progression and prognosis of LUAD”.

In the original article, there was an error in the section **Discussion**, Paragraph 5. The word “YTHDF2” in this sentence is incorrect: “Finally, Kaplan-Meier curve analysis showed that LUAD patients with high YTHDF2 expression had a worse prognosis. We believe that the cancer promoting effect of NPM1 gene is related to the modification of m6A, which may affect the methylation level of LUAD through its association with YTHDF2, and ultimately affect the progression of LUAD”.

A correction has been made to the above sentence, as follows: “Finally, Kaplan-Meier curve analysis showed that LUAD patients with high HNRNPC expression had a worse prognosis. We believe that the cancer promoting effect of NPM1 gene is related to the modification of m6A, which may affect the methylation level of LUAD through its association with HNRNPC, and ultimately affect the progression of LUAD”.

The authors apologize for these errors and state that they does not change the scientific conclusions of the article in any way. The original article has been updated.

## Publisher’s Note

All claims expressed in this article are solely those of the authors and do not necessarily represent those of their affiliated organizations, or those of the publisher, the editors and the reviewers. Any product that may be evaluated in this article, or claim that may be made by its manufacturer, is not guaranteed or endorsed by the publisher.

## Supplementary Material

Supplementary Table 1 NPM1 co-expressed genes.

Supplementary Table 2 The GO and KEGG enrichment analysis of NPM1 co-expression genes.

