# Supplementary material for: NPM1 Is a Prognostic Biomarker Involved in Immune Infiltration of Lung Adenocarcinoma and Associated With m6A Modification and Glycolysis
Source: Front Immunol. 2021 Jul 16;12:724741. doi: 10.3389/fimmu.2021.724741 (PMC8324208; doi:10.3389/fimmu.2021.724741)
Supplement: Supplementary file 4 [file DataSheet_1.docx]

Supplementary Material

# Supplementary Methods

## Cell Lines and Cell Culture Reagents

Human LUAD cell lines A549 and normal human lung epithelial cells line BEAS-2B were obtained from the American Type Culture Collection (Manassas, VA, USA). The cells were maintained in DMEM high glucose medium (Hyclone, Logan, UT, USA) supplemented with 10% FBS (Gibco, USA) and 1% antibiotics (penicillin-streptomycin, Gibco, USA).

## RNA Extraction and qRT-PCR

The implementation method refers to previous study. Total RNA was isolated from cells using Trizol reagent (Invitrogen, Carlsbad, CA, USA). Use Prime Script RT reagent kit (Takara, Dalian, China) for reverse transcription, and then use SYBR Prime Script RT PCR kit (Takara, Dalian, China) for qRT-PCR. Use GAPDH as an internal reference and use the 2^-△△Ct^ method to calculate the results. NPM1 primer sequences: forward primer CGCTGTGGAGGAAGATGCAG and reverse primer GGCAGACCGCTTTCCAGATA. GAPDH primer sequences: forward primer GGAGCGAGATCCCTCCAAAAT and reverse primer GGCTGTTGTCATACTTCTCATGG.

## Patients

A retrospective review of 40 LUAD patients who received ^18^F-FDG PET/CT scans at Taihe Hospital from February 2018 to January 2019. Inclusion criteria were as follows: (a) no biopsy, radiation or chemotherapy prior to PET/CT scan; (b) Surgery within 4 weeks after PET/CT scan; (c) Surgical resection of the tissue proved by pathology to be LUAD; (d) Complete case records.

## Immunohistochemistry and analysis

IHC staining was performed on LUAD patients undergoing PET/CT scans to assess NPM1 expression levels. The LUAD tissue and the paracarcinoma tissues were prepared into 3 μm paraffin sections and incubated with mouse monoclonal antibodies of NPM1 (1:100, Abcam, USA) at 4℃ overnight in a refrigerator. The sections were coupled with the goat anti-mouse IgG-HRP secondary antibody (1:2000, Abcam, USA) at room temperature for 1.5 h, then each incubated section was stained with DAB reagent, and finally counterstained with hematoxylin.

IHC staining scores of NPM1 were assessed by two experienced observers. IHC score of tumor cells was 0-3: 0, negative; 1, weak; 2, medium; 3, strong.

# Supplementary Tables

## Supplementary Table 1. NPM1 co-expressed genes

## Supplementary Table 2. The GO and KEGG enrichment analysis of NPM1 co-expression genes

## Supplementary Table 3. NPM1 GSEA in LUAD
